# Validating the Unmind Index as a measure of mental health and wellbeing among adults in USA, Australia, and New Zealand

**DOI:** 10.1371/journal.pone.0287215

**Published:** 2023-11-02

**Authors:** Eoin Travers, Bao Sheng Loe, Luning Sun, Heather Bolton

**Affiliations:** 1 Unmind Ltd, London, United Kingdom; 2 University of Cambridge, Cambridge, United Kingdom; Mokpo National University, REPUBLIC OF KOREA

## Abstract

**Background:**

The Unmind Index is a 26-item, 7-subscale measure of mental health and wellbeing designed for use on the Unmind digital workplace mental health platform. The Unmind Index was developed and validated in the UK but is used internationally. This paper reports further psychometric validation of this measure for use in USA, Australia, and New Zealand (ANZ).

**Methods:**

Participants in four countries completed the Unmind Index and a battery of existing measures. In Study 1 (N = 770), we validated the Unmind Index separately in USA and in ANZ. In Study 2 (N = 600), we used multiple group confirmatory factor analysis to test the measurement invariance of the Unmind Index across the UK, USA, and ANZ.

**Results:**

Study 1 establishes the factor structure, reliability, convergent and discriminant validity, and measurement invariance by age and gender of the Unmind Index separately for USA and for ANZ. Study 2 further demonstrates measurement invariance across locations, and establishes benchmark scores by location, age, and gender.

**Conclusions:**

We conclude that the Unmind Index is valid and reliable as a measure of mental health and wellbeing in these locations.

## Introduction

Unmind is a workplace digital mental health platform that utilises tools to help users track, maintain, and improve their mental health and wellbeing [1]. One of the central features of the platform is the Unmind Index [2], a measure of mental health and wellbeing (MHWB) with seven subscales—*Calmness*, *Connection*, *Coping*, *Happiness*, *Health*, *Fulfilment*, and *Sleep—*and an overall mental health and wellbeing score. The Unmind Index is used to help users understand and monitor their MHWB and guide them towards content best suited to their particular needs. The measure uses a hierarchical or second-order factor structure, with 3–5 items nested within each subscale, and subscales nested within the overall MHWB score. Since the submission of this paper, the Unmind Index has been rebranded as the Unmind Wellbeing Tracker. We will use the original title throughout this paper for consistency with prior work.

This novel measure was needed due to limitations in existing scales. Problem-focused scales such as PHQ-9 [7] measure specific MHWB problems, but use negative language unsuited for use in an app intended for general use, and do not provide a single overall MHWB score. Conversely, holistic wellbeing scales such as WEMWBS [14] use positive language, and provide overall scores, but do not tap into specific problems such as social support or sleep quality. The Unmind Index attempts to bridge this gap.

We previously [2] described the development and validation of the Unmind Index, based on data collected from adult participants based in the UK. However, the Unmind platform is available internationally, with many users based in the USA, Australia, and New Zealand. It cannot be taken for granted that measures developed and validated in one culture and locale remain valid when used elsewhere. For this reason, we validated the Unmind Index across three countries (USA, Australia, and New Zealand) where the platform is most widely used.

There are several reasons for the Unmind Index to be validated internationally. If questions are interpreted in different ways in different cultures, a measure developed in one location may not be valid elsewhere. More subtly, a measure might be valid in multiple locations, but tap into different constructs in each location, leading to scores that should not be compared across locations. Beyond this, a measure might tap into the same construct across locations, but the distributions of scores differ, either due to differences in response biases, or underlying differences in the constructs being measured. These possibilities can be explored by testing for measurement invariance [3] across data obtained from different locations, using confirmatory factor analysis (CFA).

In this paper, we examine three locations: the United Kingdom (UK), United States of America (USA), and Australia & New Zealand (ANZ). Australia and New Zealand are treated as a single location due to their smaller populations and cultural and economic similarities. These countries have much in common culturally and economically, and form part of the *Anglo* cluster recognised in organisational scholarship [4]. Public attitudes to mental health are also similar across these countries [5]. However, to our knowledge no previous studies have assessed whether mental health measures developed in one English-speaking country are valid when used in other English-speaking locations. This paper therefore has two goals: to establish the psychometric properties of the Unmind in the USA, Australia, and New Zealand, and to explore potential differences in the hierarchical structure of MHWB across these countries and the UK.

### Outline

In Study 1, we validate the Unmind Index as a measure of mental health and wellbeing among adults in the USA, and Australia/New Zealand, including validation against a battery of existing instruments. Data collection for Study 1 took place in July 2021 in the USA but was delayed until September 2021 for Australia/New Zealand due to the imposition of COVID-19 lockdowns in July. It is therefore difficult to directly compare data between these two locations and to our original UK validation data, collected in November 2020. Therefore, in Study 2 we simultaneously collected new data in the UK, USA, and ANZ, allowing direct comparisons between the locations.

## Study 1: USA/ANZ validation

### Methods

#### Participants

*United States of America*. A nationally representative sample of 400 USA adults, stratified by age, sex, and ethnicity, was recruited using the representative sampling feature provided by the Prolific participant recruitment platform [6]. Participants were paid £3, and median completion time was 15 minutes. Data collection took place on July 13th, 2021. Data was lost from two participants due to technical issues.

Mean age was 45.1 years (SD 16.1, range 18–81), 197 (49.5%) identified their gender as female, 198 (49.7%) as male, 2 (0.6%) as non-binary, and one opted not to say. Prolific uses simplified USA census categories for ethnicity: 281 (70.6%) identified as White, 52 (13.1%) as Black, 31 (7.8%) as Asian, 19 (4.8%) as Mixed, 14 (3.5%) as “Other”, and data was missing for 1 participant. Additionally, we manually recoded participants’ self-reported ethnicity into the following categories: European (n = 262, 65.8%), African (57, 14.3%), Asian (28, 7.0%), Hispanic (27, 6.8%), Mixed (17, 4.3%), Middle Eastern (4, 1.0%), American (1, 0.3%), Native American (1, 0.3%), and 1 participant who did not specify (0.3%).

One hundred participants were randomly selected and invited to complete a follow-up questionnaire one week later, described below. 90 out of 100 participants completed this follow-up and were paid £1.10 for doing so. In this group, mean age was 45 years (SD 16, range 18–80), 43 (47.8%) identified as male and 46 (51.1%) as female. 68 participants identified as European (75.6%), 8 as African (8.9%), 6 as Asian (6.7%), 5 as Hispanic (5.6%), 2 as mixed-ethnicity (2.2%), and 1 as Middle Eastern (1.1%).

*Australia/New Zealand*. Prolific does not provide nationally representative sampling outside of the UK and USA. We therefore manually stratified our sample by age group (18–29, 30–42, 43–56, 57+) and sex (male, female), yielding eight subgroups of 50 participants each. Participants were paid £3, and median completion time was 16 minutes. Data collection began on September 30th 2021 and ran for three days. At this point, data collection was complete for all groups except for the female/57+ group (34/50 participants) and the male/57+ group (36/50). This made for a total sample size of 370 participants.

Mean age was 40.4 years (SD 15.1, range 18–84), 177 participants (47.8%) identified their gender as female, 187 (50.5%) as male, and 6 (1.8%) as non-binary. We manually recoded participants’ self-reported ethnicity into the following categories: European (n = 234, 63.2%), Asian (74, 20.0%), Australian/New Zealander (34, 9.2%), Mixed (8, 2.2%), Middle Eastern (6, 1.6%), Māori (5, 1.4%), Pacific Islander (4, 1.1%), African (2, 0.5%), Aboriginal Australian (1, 0.3%), and 2 participants who did not specify (0.5%).

One hundred participants were randomly selected and invited to complete a follow-up questionnaire one week later and were paid £1.10 for doing so. 88 of these 100 participants completed the follow-up. Mean age for this group was 42.8 years (SD 16.1, range 18–84), 45 (51.1%) identified as female, and 43 (48.9%) as male. 55 participants identified as European (62.5%), 20 as Asian (22.7%), 9 as ANZ (10.2%), 1 as Māori (1.1%), 1 as Pacific Islander (1.1%), 1 as Mixed (1.1%), and 1 participant who did not specify (1.1%).

*Ethics*. Both studies reported in this manuscript received ethical approval from the University of Cambridge (Judge Business School Departmental Ethics Review Group, approval number 20–061), and all participants provided informed consent prior to taking part.

#### Measures & hypotheses

Participants completed the 26-item Unmind Index and a battery of established mental health and wellbeing measures, and provided demographic information.

In the Unmind Index, participants are shown the prompt “*During the past two weeks I have*…*”*, followed by the item text (e.g. “*been feeling cheerful or bright in my mood”*) and are asked to rate how often each item applies to them on a 6-point Likert scale from “*No days*” (0) to “*Every day*” (5). Items were presented in random order.

The existing measures of mental health and personality used in this study, and the Unmind Index subscales they were expected to correlate with, are summarised in Table 1. We expected the PHQ-8 [7] to correlate negatively with the *Happiness* subscale, GAD-7 [8] to correlate negatively with *Calmness*, the HADS [9] anxiety subscale to correlate negatively with *Calmness*, and HADS depression negatively with *Happiness*, the Perceived Stress Scale [10] to correlate negatively with *Coping*, the PROMIS sleep disturbance short form [11] to correlate negatively with *Sleep*, the PROMIS-10 [12] physical health subscale to correlate positively with *Health*, and Brief Inventory of Thriving [13] to correlate positively with *Fulfilment*. The Warwick-Edinburgh Mental Wellbeing Scale [14] was expected to correlate positively with the Unmind Index overall score. To establish the discriminant validity of the Unmind Index, we also included the Ten-Item Personality Inventory [15], a brief scale that measures individual differences in the “big five” personality traits (extraversion, agreeableness, conscientiousness, emotional stability, and openness to experiences).

**Table 1:** Established measures used to test concurrent and discriminant validity of the Unmind Index. Reliability estimates are averages across USA and ANZ samples.

Measure	Label	Domain	N	Subscales	K	Score Range	Reliability (α)	Unmind Index Subscale
Patient Health Questionnaire 8	PHQ-8	Depression	8	-	4	0–24	.91	Happiness
General Anxiety Disorder 7	GAD-7	Anxiety	7	-	4	0–21	.93	Calmness
Hospital Anxiety and Depression Scale	HADS	Anxiety; Depression	14	Anxiety Depression	4	0–21	.87 (Anxiety) .84 (Depression)	Calmness (Anxiety) Happiness (Depression)
Perceived Stress Scale	PSS	Stress	10	-	5	0–40	.91	Coping
PROMIS Sleep Disturbances–Short Form	PROMIS-Sleep	Sleep disorders	9	-	5	0–45	.93	Sleep
UCLA Loneliness Scale V3	ULS-v3	Loneliness and social isolation	20	-	4	20–80	.95	Connection
PROMIS Global Health	PROMIS-10	Mental, physical, and overall health	10	Metal health Physical health Combined health	5^*†*^	4–20 (subscales); 10–50 (combined)	.85 (Mental) .70 (Physical) .65 (Combined)	Health (PROMIS Physical)
Brief Inventory of Thriving	BIT	Positive wellbeing	10	-	5	1–5	.94	Fulfilment
Warwick-Edinburgh Mental Wellbeing Scale	WEMWBS	Overall wellbeing	14	-	5	14–70	.95	Total Score
Ten-Item Personality Inventory	TIPI	Big Five personality traits	10	Extraversion Agreeableness Consc. Emot. Stability Openness	7	2–14	.73 (Extraversion) .34 (Agreeableness) .65 (Consc.) .75 (Emot. stability) .36 (Openness)	None (control measure)

*Note*: N = Number of items; K = Number of response options; Consc. = Conscientiousness; Emot. Stability = Emotional stability.

#### Analyses

All statistical analyses were performed in R (v4.0.3) [16]. Unless otherwise noted, all analyses were performed separately for USA participants and ANZ participants. Direct comparisons between locations are reported in Study 2.

**Fig 1:**
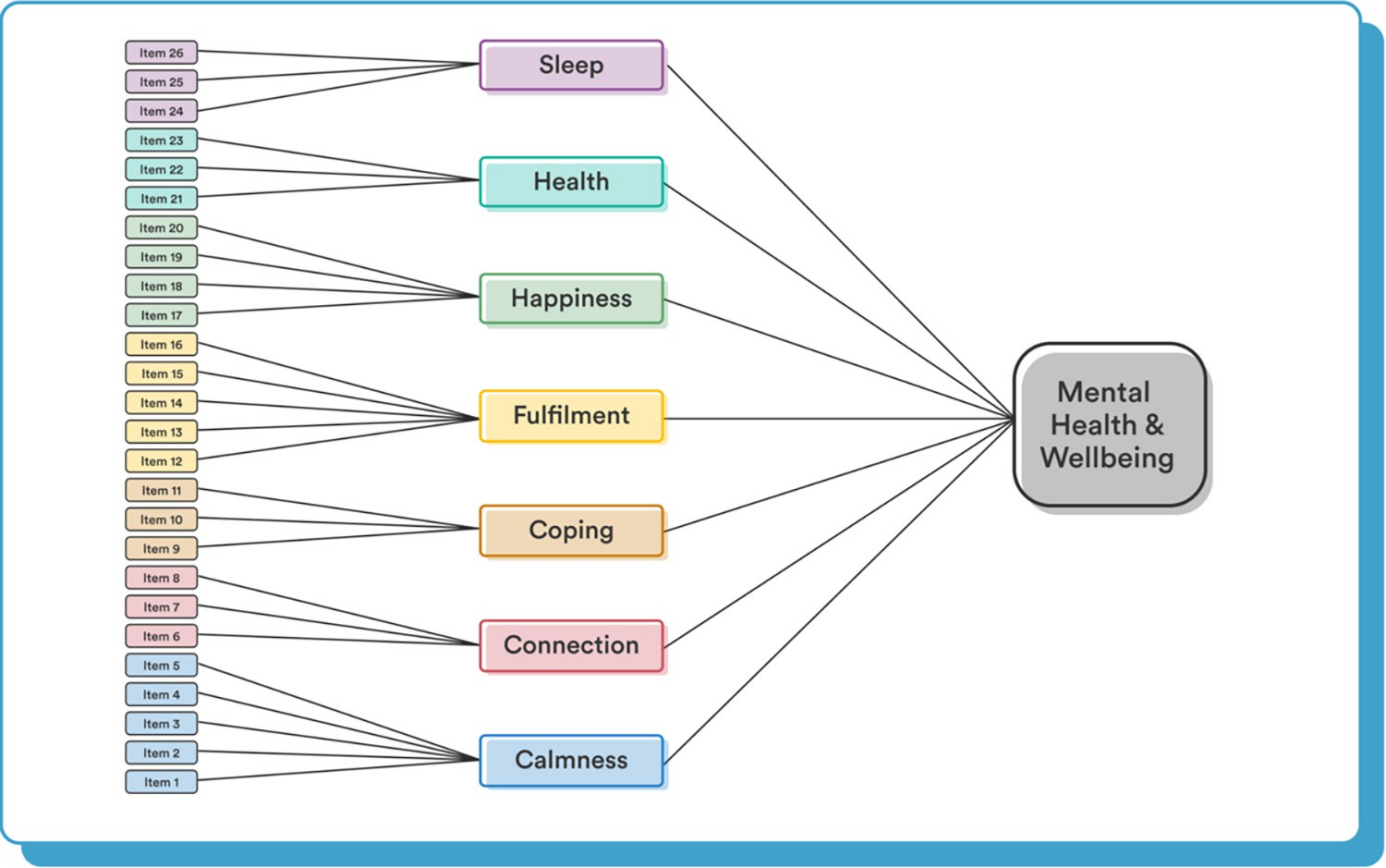
The second-order factor structure used for the Unmind Index.

*Confirmatory factor analysis*. The factor structure of the Unmind Index was tested through confirmatory factor analysis (CFA), using the lavaan package for R [17], with maximum-likelihood estimation. As each question has six possible response options, and so cannot meet assumptions of normality, we used robust Huber-White standard errors and fit statistics. Our previous work [2] showed that a second-order factor structure (Fig 1) provided a good fit for Unmind Index data collected from UK participants, and this structure is used to calculate Unmind Index scores on the Unmind platform. In this, every item loads on to one of the seven Unmind Index subscales, *Happiness*, *Sleep*, *Coping*, *Calmness*, *Health*, *Connection*, and *Fulfilment*, and each subscale loads onto the general *Mental Health and Wellbeing* factor. A bifactor model was also considered in our previous work [2], but is not discussed further here. To explore relationships between subscales, we also fit a correlated-factors model, where the subscales do not load onto a general factor, but correlations between subscales are estimated directly. All latent factors were standardized to have a variance of 1.

Model fit was evaluated using several indices: the Comparative Fit Index (CFI), Tucker-Lewis Index (TLI), Root Mean Square Error of Approximation (RMSEA), and the Standardized Root Mean Residual (SRMR). A model fit > .90 was considered acceptable for both CFI and TLI, and > .95 considered good [18]. For RMSEA and SRMR, a value between .06-.08 was considered an acceptable fit, while a value < .06 was considered a good fit [19]. To identify potential causes of poor model fit, we inspected correlation residuals between each pair of items—mismatches between the correlations implied by the fitted model and those observed in the data. Correlation residuals greater than 0.1 in absolute value were identified as notable departures [20].

*Test-retest reliability*. Test-retest reliability was estimated for participants who completed the one-week follow-up questionnaire, using two-way consistency intra-class correlation coefficients, *ICC(C*, *1)*.

*Internal consistency*. To determine internal consistency of the Unmind Index, we computed Cronbach’s α. As the tau-equivalence assumption of α are rarely met in practice we also calculated coefficient omega [21] as an indicator of internal consistency.

*Convergent and discriminant validity*. The existing measures of mental health and personality used in this study, and the Unmind Index subscales they were expected to correlate with, are summarised in Table 1. Pearson correlations were computed between each existing measure and Unmind Index scores and adjusted for reliability (dis-attenuated) using the Cronbach’s α estimates for each measure. In cases where the correlation between measures was predicted to be negative, for instance between Unmind Index *Happiness* scores and PHQ-8, the sign of the correlation is reversed to be positive for clarity. Unmind Index subscale scores were calculated by averaging responses within each subscale after reverse-scoring, and total Unmind Index scores as the average of the seven subscale scores.

Given the strong associations typically found between various mental health measures [22], we assessed convergent validity by checking that the pattern of correlations of Unmind Index subscale scores with the relevant existing measures (e.g. *Happiness* and PHQ-8) are a) strong, and b) stronger than the correlation with less relevant existing measures (e.g. *Happiness* and GAD-7). Discriminant validity was similarly assessed by checking that correlations between Unmind Index subscales and TIPI personality subscales are weak, and weaker than correlations between the Unmind Index and mental health measures.

*Measurement invariance*. We used multiple-group CFA to test the *measurement invariance* of the Unmind Index across age and gender groups. This allows us to test that factor structures are consistent across groups, that loadings are consistent, and that scores are not biased by differences in response to individual items. These conditions must be met for scores to be validly compared across groups. Median participant age was 44 years in USA, and 39 years in ANZ, and participants were classed as either older or younger than the median in each location. 9 participants who responded “Non-binary”, “Other” or “Prefer not to say” when asked about their gender identity were excluded from the gender invariance analysis. Measurement invariance between locations is tested in Study 2.

Measurement invariance was tested in accordance with the steps outlined by Millsap [3]. We began by fitting a *configural invariance* model, where both groups have the same factor structure, but all parameter values are allowed to differ between groups. Achieving a good model fit here indicates that both groups have the same overall factor structure. We then compared this model to a *weak/metric invariance* model, where first- and second-level factor loadings are constrained to be equal across groups. If this constraint does not appreciably reduce model fit, we can conclude that factor weights are consistent across groups. Lastly, we fitted a *strong/scalar invariance* model, where item intercepts are also constrained to be equal, but factor means are allowed to differ between groups. If this does not show a poorer fit than the weak invariance model, we can conclude that item intercepts are equivalent across groups. In other words, any differences in factor scores are not driven by group differences on specific items. It is only appropriate to compare factor scores across groups if this third condition is met.

To compare model fits, we calculate CFI and Bayesian Information Criteria (BIC) for each model. We consider a constrained model to have worse fit than the unconstrained alternative if CFI is more than 0.01 points lower for the constrained model [23], or if BIC is higher. An increase or a reduction of less than 0.01 points in CFI, along with a decrease in BIC, constitutes evidence for invariance.

The analyses described above test whether measurement invariance holds for the 26-item Unmind Index as a whole—that is, whether the data is better accounted for by a model where all factor loadings and item intercepts are constrained to be equal across groups, versus a model where all loadings and intercepts are allowed to vary. Similar analyses of individual subscales are reported in S1 File.

### Results

#### Model fit

Table 2 shows CFA model fit indices for the second-order factor model, fit to data from USA and ANZ, indicating acceptable fit to the data in both locations.

**Table 2:** Fit indices for the second-order factor model fit to data from USA and Australia/New Zealand (ANZ).

Location	χ^2^	DF	SRMR	RMSEA [90% CI]	CFI	TLI
USA	819.6	292	.068	.074 [.068, .080]	.917	.907
ANZ	755.3	292	.065	.070 [.064, .076]	.922	.913

Standardised item-to-factor loadings and residual variance estimates for each item are shown in Table 3. Means and standard deviations for each subscale and for the overall score are shown int Table 4. Correlations between subscales are shown in Table 5.

**Table 3:** Standardised item-to-factor loadings and residual variance for the second-order factor model, fit to data from USA and ANZ.

		USA		ANZ	
Item	Factor	Loading	σ^2^	Loading	σ^2^
Felt a sense of accomplishment	Fulfilment	.83 (±.02)	.31	.80 (±.02)	.36
Been feeling cheerful or bright in my mood	Fulfilment	.82 (±.02)	.33	.82 (±.03)	.33
Felt like I am leading a fulfilling life	Fulfilment	.84 (±.02)	.30	.83 (±.02)	.31
Been feeling good about myself as a person	Fulfilment	.86 (±.02)	.25	.87 (±.02)	.25
Felt that I am growing positively as a person	Fulfilment	.79 (±.03)	.38	.80 (±.03)	.37
Felt appreciated by others	Connection	.83 (±.03)	.32	.78 (±.03)	.39
Felt connected to people around me	Connection	.83 (±.03)	.31	.85 (±.03)	.28
Felt like I have warm and trusting relationships with others	Connection	.82 (±.03)	.34	.86 (±.02)	.26
Felt disappointed in myself	Happiness	.86 (±.02)	.26	.84 (±.02)	.30
Found it hard to motivate myself to engage with everyday tasks	Happiness	.79 (±.02)	.37	.74 (±.03)	.45
Had little interest in people or activities that I used to enjoy	Happiness	.68 (±.04)	.54	.69 (±.04)	.53
Tended to get stuck in a cycle of negativity in my head	Happiness	.89 (±.02)	.21	.83 (±.03)	.32
Been feeling down or sad in my mood	Happiness	.87 (±.02)	.23	.85 (±.02)	.27
Felt like I am in a good state of health	Health	.87 (±.03)	.25	.87 (±.02)	.25
Been managing my health well	Health	.81 (±.03)	.34	.86 (±.02)	.26
Felt that my physical health is not as good as I’d like it to be (given my age/life circumstances)	Health	.63 (±.05)	.61	.64 (±.04)	.59
Been able to proactively manage my stress day to day	Coping	.75 (±.03)	.43	.80 (±.03)	.36
Felt confident that I can handle problems that come my way	Coping	.84 (±.02)	.29	.87 (±.03)	.25
Felt able to cope if something unexpected happens	Coping	.72 (±.04)	.49	.78 (±.03)	.40
Felt satisfied with my sleep	Sleep	.89 (±.02)	.20	.89 (±.02)	.22
Had trouble falling or staying asleep, or waking up too early	Sleep	.73 (±.03)	.47	.73 (±.04)	.47
Slept well, all things considered (e.g. such as caring for young children at night, snoring partner, shift work, etc)	Sleep	.86 (±.03)	.26	.88 (±.03)	.23
Found it hard to stop (or control) worrying	Calmness	.88 (±.02)	.22	.86 (±.02)	.26
Had difficulty switching off	Calmness	.76 (±.03)	.42	.70 (±.04)	.51
Noticed that my body has been tense	Calmness	.77 (±.03)	.40	.70 (±.04)	.51
Worried that bad things might happen to me or others close to me	Calmness	.74 (±.03)	.45	.70 (±.03)	.51

**Table 4:** Mean and standard deviations of raw scores for each subscale and total score (from 0 to 5), and standardised loadings (± standard errors) of each subscale onto the second-order mental health and wellbeing factor.

	USA			ANZ		
	Mean	SD	Loading	Mean	SD	Loading
Calmness	2.93	1.42	.81 (±.04)	2.80	1.28	.74 (±.04)
Connection	3.33	1.16	.76 (±.04)	2.99	1.16	.80 (±.03)
Coping	3.42	1.08	.92 (±.02)	3.13	1.11	.92 (±.02)
Fulfilment	3.10	1.18	.92 (±.02)	2.70	1.14	.93 (±.02)
Happiness	2.98	1.42	.87 (±.03)	2.76	1.30	.87 (±.02)
Health	2.91	1.27	.82 (±.03)	2.47	1.27	.85 (±.02)
Sleep	2.91	1.41	.67 (±.04)	2.64	1.35	.59 (±.04)
Total	3.08	1.03		2.78	0.98	

**Table 5:** Pearson correlation coefficients (± standard errors) for correlations between Unmind Index subscale scores. Coefficients for USA are shown above the diagonal, and values for ANZ below.

	Total	Happiness	Calmness	Coping	Sleep	Health	Connection	Fulfilment
Total	-	.87	.81	.83	.73	.77	.74	.86
Happiness	.87	-	.83	.70	.50	.61	.53	.68
Calmness	.77	.79	-	.64	.53	.52	.43	.55
Coping	.84	.66	.57	-	.52	.57	.56	.72
Sleep	.69	.47	.49	.45	-	.49	.46	.56
Health	.80	.62	.51	.67	.48	-	.52	.66
Connection	.76	.58	.39	.61	.41	.54	-	.73
Fulfilment	.85	.69	.48	.76	.47	.66	.75	-

**Fig 2:**
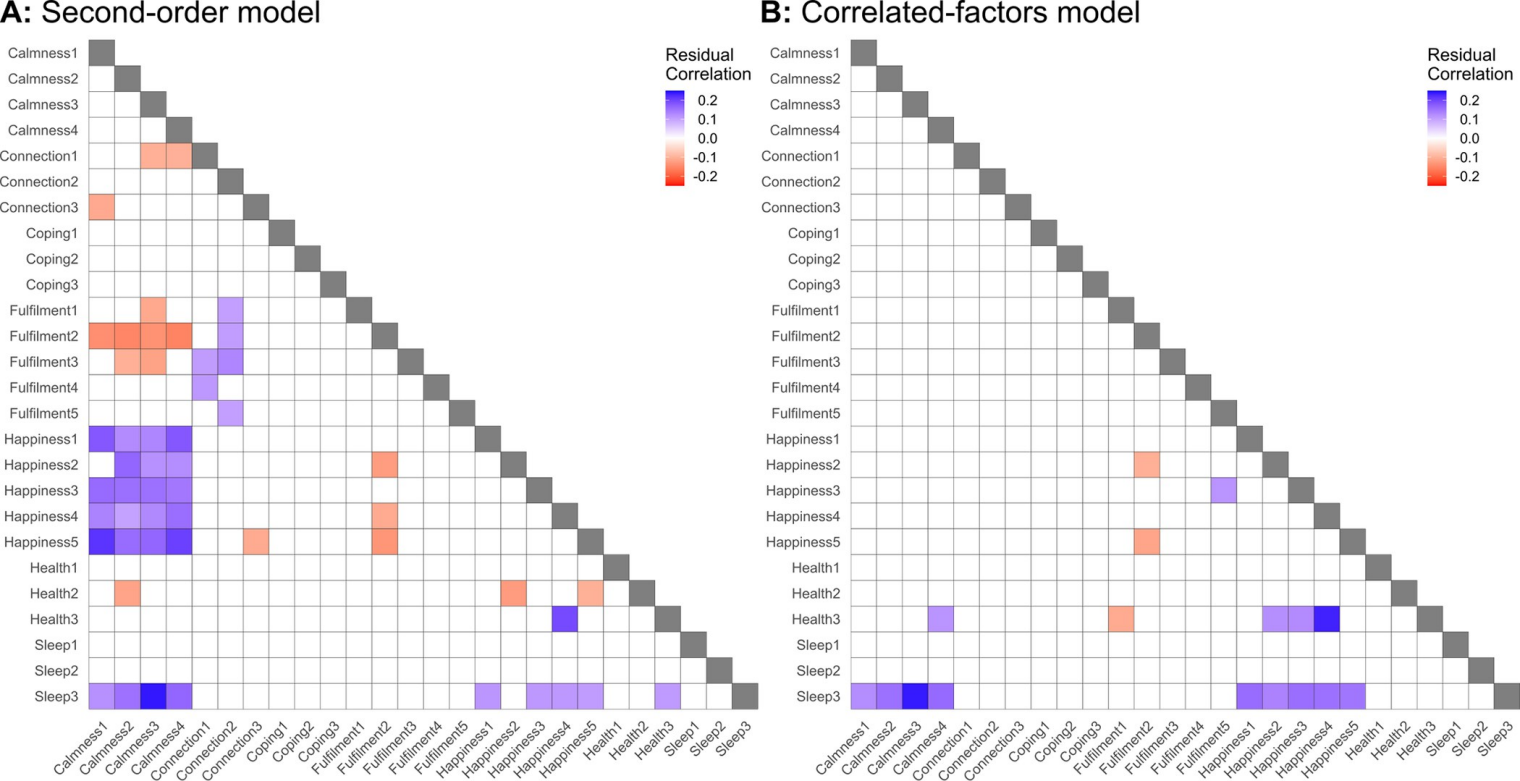
Correlation residuals greater than 0.1 in absolute value for the second-order (**A**) and correlated-factors (**B**) CFA models, for the USA sample. Large residuals reflect ways in which a model fails to fully capture the correlation between pairs of items.

Correlation residuals greater than 0.1 in absolute value for the second-order model fit to USA data are shown in Fig 2A. Corresponding residuals for ANZ data are shown in S1 File. These show that the second-order model could not fully explain the positive correlations between items in the *Happiness* and *Calmness* subscales. Model fit was substantially improved in the correlated factors model, which explicitly models correlations between subscales: χ^2^(278) = 563.4, SRMR = .048, RMSEA = .056 (90% CI [.049; .063]), CFI = .955, TLI = .948. Correlation residuals for this model (Fig 2B) show that the associations between *Happiness* and *Calmness* items are captured by the direct correlation between these two factors, but substantial unexplained correlations remain between the third *Sleep* item (“*Had trouble falling or staying asleep*, *or waking up too early*”) and the *Happiness* and *Calmness* items. Allowing this item to load onto the *Happiness*, *Calmness*, and *Sleep* factors yields excellent model fit, χ^2^(276) = 510.7, SRMR = .041, RMSEA = .051 (90% CI [.044; .058]), CFI = .963, TLI = .957. Importantly, factor scores estimated from the second-order model by least squares regression are very highly correlated with those estimated from this final model, r > .97, indicating that the less-than-perfect fit of the second-order model does not reduce its usefulness for scoring users’ responses. Consistent results were obtained for the ANZ data, reported in S1 File.

#### Consistency and reliability

Estimates of internal consistency and test-retest reliability are shown in Table 6. In both the USA and ANZ, internal consistency and test retest reliability (ICC(C, 1)) were good for all subscales and for the total score. There were no clear differences in consistency or reliability between locations.

**Table 6:** Internal consistency (Cronbach’s α and McDonald’s ω) and test-retest reliability, ICC(C,1) for subscales and total scores, by location. Values in brackets show 95% confidence intervals.

	USA			ANZ		
	Consistency		Test-Retest	Consistency		Test-Retest
Scale	α	ω	ICC(C,1)	α	ω	ICC(C,1)
Happiness	.91	.91	.89 [.84, .93]	.89	.89	.81 [.72, .87]
Sleep	.86	.86	.84 [.76, .89]	.86	.86	.83 [.76, .89]
Coping	.81	.81	.78 [.68, .85]	.85	.86	.78 [.69, .85]
Calmness	.87	.87	.83 [.75, .88]	.83	.83	.80 [.71, .86]
Health	.79	.80	.81 [.73, .87]	.82	.82	.76 [.65, .84]
Connection	.86	.86	.82 [.73, .87]	.87	.87	.83 [.75, .89]
Fulfilment	.92	.92	.87 [.81, .91]	.91	.91	.84 [.77, .89]
Index Total		.91	.91 [.87, .94]		.91	.90 [.85, .93]

#### Convergent and discriminant validity

**Fig 3:**
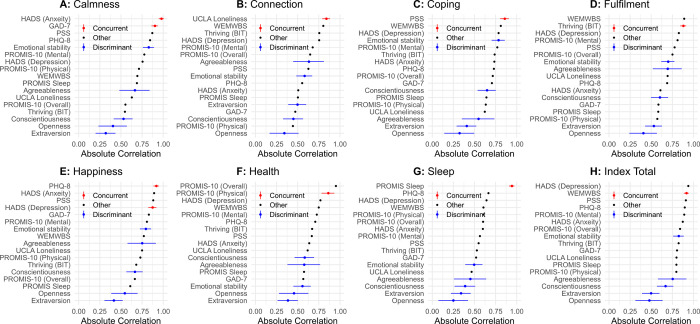
Dis-attenuated absolute correlation coefficients between Unmind Index scores and existing measures for the USA sample. Values in red show correlations with mental health and wellbeing measures predicted to correlate most strongly with the Unmind Index subscale in question. Values in blue show personality measures, which were expected to correlate most weakly with all scales. Error bars show standard error.

*USA*. Correlations between Unmind Index subscales and existing measures for USA participants, corrected for attenuation, are shown in Fig 3. Correlations without disattenuation are reported in S1 File and show consistent results unless otherwise noted.

In general, Unmind Index subscales were most strongly associated with the expected measures of mental health and wellbeing, slightly less associated with other measures of mental health and wellbeing, and only weakly associated with personality traits. However, there were several exceptions. The association between the *Fulfilment* subscale and the WEMWBS, a general measure of wellbeing, was as strong as that between *Fulfilment* and the Brief Inventory of Thriving, the measure expected to correlate most strongly with this subscale. Although the *Happiness* subscale was as expected most strongly associated with PHQ-8 scores, the association with the HADS depression subscale was weaker than expected, and was of the same magnitude as the association with the HADS anxiety subscale, GAD-7, and PSS. This suggests that the *Happiness* subscale measures a construct related to depression, anxiety, and stress, rather than depression alone. The *Health* subscale was most strongly associated with PROMIS-10 combined (physical and mental) health scores than PROMIS physical health scores. Finally, TIPI emotional stability scores and agreeableness scores were both moderately or strongly associated with scores on the Unmind Index *Calmness*, *Connection*, *Coping*, *Fulfilment*, and *Happiness* subscales.

**Fig 4:**
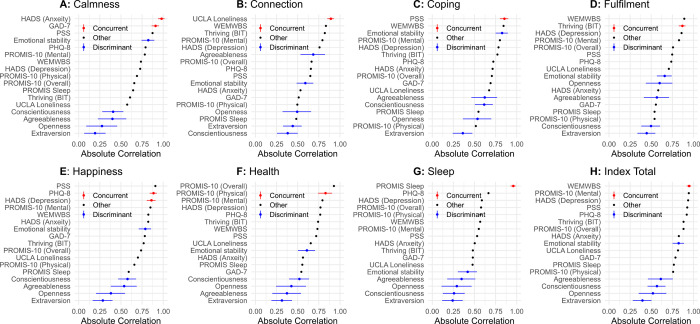
Dis-attenuated absolute correlation coefficients between Unmind Index scores and existing measures for the ANZ sample.

*ANZ*. Equivalent correlations for ANZ participants are shown in Fig 4, with correlations without disattenuation reported in S1 File. The overall pattern of correlations was as expected, but there were once again some exceptions. As was the case for USA participants, the *Fulfilment* subscale was more strongly associated with WEMWBS scores, and *Health* subscale scores more strongly associated with PROMIS-10 overall health scores, than expected. TIPI emotional stability and agreeableness scores were moderately to strongly associated with several Unmind Index subscales. Unmind Index *Happiness* scores were most strongly associated with PSS scores, followed by PHQ-8, the HADS depression subscale, and PROMIS-10 mental health subscale.

#### Invariance

Measurement invariance results by age and gender, for USA and ANZ participants, are reported in Table 7. For all comparisons, BIC scores were lowest for the strong invariance model, and CFI values were superior, or inferior by less than -.01, for the strong invariance model. We therefore conclude that the Unmind Index shows measurement invariance by age and by gender, both in the USA and ANZ. CFI values were generally below the “acceptable” cut-off of 0.9. However, as discussed above, this largely reflects the inability of the second-order factor model to account for correlations between the *Calmness* and *Happiness* subscales, and factor scores estimated from this slightly mis-specified model correlate almost perfectly with scores estimated from a model that directly models factor correlations. Finally, we found that Unmind Index scores were higher for older participants, and for male participants. These patterns are consistent with the results of Study 2, presented in detail below.

**Table 7:** Measurement invariance results by age and gender, within USA and ANZ.

Invariance factor	Location	CFI			BIC		
		Configural	Weak	Strong	Configural	Weak	Strong
Age	USA	0.890	.891 (Δ = +.000)	.885 (Δ = -.005)	175	50 (Δ = -125)	0 (Δ = -50)
	ANZ	0.897	.896 (Δ = -.000)	.891 (Δ = -.006)	171	49 (Δ = -122)	0 (Δ = -49)
Gender	USA	0.900	.899 (Δ = -.001)	.898 (Δ = -.002)	194	75 (Δ = -119)	0 (Δ = -75)
	ANZ	0.907	.909 (Δ = +.002)	.909 (Δ = -.000)	226	88 (Δ = -137)	0 (Δ = -88)

Measurement invariance results for each of the seven Unmind Index subscales by age and gender are reported in S1 File. By gender, all scales showed evidence of strong measurement invariance in both USA and ANZ. By age group, there was some evidence of violation of measurement invariance for the *Coping* subscale for USA participants, and the *Calmness*, *Fulfilment*, and *Happiness* subscales for ANZ participants. Of these violations, only one was replicated in Study 2: weak but not strong invariance for the *Fulfilment* subscale by age groups (reported in S1 File).

### Discussion

These results indicate that the second-order factor structure of the Unmind Index provides an acceptable fit to data from USA and ANZ. However, model fit was not ideal, and inspection of the correlation residuals indicated that the *Happiness* and *Calmness* subscales are more strongly correlated than would be expected given the second-order model. This is consistent with the results reported in our previous UK validation study [2]. These subscales capture symptoms associated with depression and anxiety, respectively. Given the known associations between depression and anxiety [24], it is unsurprising that the subscales should be more correlated with each other than they are with other subscales such as *Sleep* or *Fulfilment*. Although these subscales are strongly correlated, we believe that Unmind’s users are best-served by maintaining two distinct subscales in second-order factor structure, since the subjective experiences associated with depression symptoms and anxiety symptoms are quite different, and the Unmind platform provides distinct resources for addressing each set of symptoms. This is in line with diagnostic theory and clinical practice [24]. Our analyses also showed that our decision to use a second-order structure rather than the better-fitting correlated-factors structure does not distort the scores obtained on each subscale, as scores from the two structures are almost perfectly correlated.

Also in line with our previous UK validation study [2], the current results also indicate that the Unmind total score shows excellent reliability, and subscale scores show good reliability. The current convergent validity results are also broadly consistent with our predictions, and with the results obtained in the UK sample, with a few exceptions described above. These exceptions may reflect international heterogeneity for some of the constructs in question, although it is not clear if these differences in the behaviour of the Unmind Index across locations, or differences in the construct validity of other measures, such as the Perceived Stress Scale. Unfortunately, at present few measures are separately validated for use across different English-speaking locations. We found that several subscales were estimated to correlate more strongly than was expected with personality traits assessed by TIPI, in particular the *emotional stability* and *agreeableness* traits. However, these correlation estimates are to some degree inflated by the low reliability of the TIPI measures (α = .75 and .34 respectively), which are taken into account when estimating the disattenuated correlation coefficients. It should also be noted that emotional stability has previously been shown to correlate strongly with existing measures of mental health problems [25]. Finally, the Unmind Index as a whole displayed strong measurement invariance by age and by gender in both USA and ANZ. Invariance results for the subscales are discussed below.

## Study 2: Invariance by location

In Study 1, we established that the Unmind Index is a valid and reliable measure of mental health and wellbeing in USA and in ANZ. We previously established the same conclusions in the UK [2]. Our next goal was to establish if Unmind Index scores can be validly compared across these locations—that is, if it shows measurement invariance across locations—and if so, to compare scores obtained in each location and establish appropriate benchmarks for standardised scoring.

Given the different times at which the data described in Study 1 and in our original UK validation [2] were collected, it would not be appropriate to directly compare results across these datasets. For this reason, we decided to obtain a new dataset of participants in the UK, USA, and ANZ, collected concurrently.

### Methods

#### Participants

600 participants were recruited using the Prolific platform, and the sample was stratified by location (UK, USA, and ANZ), age (18–42 years, 43 and over) and sex (male and female) into twelve subgroups of 50 participants each. Testing took place on November 8th, 2021. Detailed characteristics of this sample are reported in S1 File.

#### Measures

Participants were presented with the Unmind Index, followed by the demographic questions from Study 1.

#### Analyses

*Measurement invariance*. To establish measurement invariance across locations, we used multiple-group confirmatory factor analysis, fitting the second-order factor model with parameters allowed to vary or constrained to be equal between locations, as described above. We report results from omnibus tests comparing models in which parameters are constrained to be equal in all three locations to models in which parameters are allowed to vary. All measurement invariance analyses were also conducted for each of the seven Unmind Index subscales individually, using single-factor CFA models.

*Group comparisons*. To explore differences in Unmind Index scores by location, age, and gender, we calculated the mean, standard deviation, and standard errors of scores within each subgroup. For this purpose, we split participants into four age groups: 18–25, 26–40, 41–50, and 51–84. Due to the small numbers involved, participants who reported genders other than “male” or “female” are excluded from these analyses.

Complete tables of these benchmark statistics for each Unmind Index subscale are reported in S1 File. To visualise these patterns, we treat age as a continuous variable and plot loess-smoothed estimates using the ggplot2 package for R [26]. Finally, to summarise these patterns, we fit a linear model with location, gender, and age as predictors. Age was centred on the mean of 40 years and divided by 10, “female” was coded as the baseline for gender, and “UK” as the baseline for location. As a result, the intercept term is an estimate for UK female participants aged 40, and the remaining coefficients indicate how scores differ from this reference value, with the age coefficient reflecting the change in scores for a 10-year increase in age. For clarity, we show plots and report regression results for only total Unmind Index scores below. Full results are reported in S1 File. As these analyses are exploratory, we do not report p-values for hypothesis tests.

### Results

#### Invariance

Measurement invariance results are summarised in Table 8. For the full Unmind Index CFI for the configural invariance model, where all parameters are allowed to vary across locations, was .903. This value is acceptable, and consistent with the results of Study 1 (CFI = .910 for USA, .917 for ANZ). Constraining factor loadings to be equal across locations in the weak invariance model increased CFI by .001 to .904, and additionally constraining item intercepts to be equal in the strong invariance model reduced CFI by only .003 to .902. Consistent with this, BIC was 68 points lower for the strong invariance model than the weak model, and 130 points lower for the weak model than the configural model. Therefore, we conclude that the Unmind Index displays strong measurement invariance across locations, and so scores can be compared across locations.

**Table 8:** Tests of measurement invariance for the Unmind Index and its individual subscales across locations (UK, USA, and ANZ). Results indicate that strong measurement invariance holds for the full Unmind Index, and individually for each subscale.

	Comparative Fit Index			Bayesian Information Criterion		
Scale	Configural	Weak	Strong	Configural	Weak	Strong
Unmind Index	.903	.904 (Δ = +.001)	.902 (Δ = -.003)	198	68 (Δ = -130)	0 (Δ = -68)
Fulfilment	.974	.977 (Δ = +.003)	.975 (Δ = -.002)	89	39 (Δ = -50)	0 (Δ = -39)
Connection	1.000	.999 (Δ = -.001)	1.000 (Δ = +.001)	44	23 (Δ = -21)	0 (Δ = -23)
Happiness	.992	.994 (Δ = +.002)	.992 (Δ = -.002)	87	40 (Δ = -47)	0 (Δ = -40)
Health	1.000	.994 (Δ = -.006)	.991 (Δ = -.003)	37	20 (Δ = -17)	0 (Δ = -20)
Coping	1.000	.997 (Δ = -.003)	.990 (Δ = -.007)	35	16 (Δ = -19)	0 (Δ = -16)
Sleep	1.000	1.000 (Δ = +.000)	.996 (Δ = -.004)	38	15 (Δ = -24)	0 (Δ = -15)
Calmness	.972	.976 (Δ = +.004)	.968 (Δ = -.008)	60	25 (Δ = -36)	0 (Δ = -25)

Similar results were found for each subscale (Table 8), with all CFI values ≥ .972 for configural invariance models, no changes in CFI ≤ -.008 observed when constraining parameters by location, and the strong invariance model obtaining the lowest BIC for all subscales. Therefore, we conclude that Unmind Index subscale scores also display strong measurement invariance, and can be compared across locations.

#### Benchmarks and group comparisons

**Fig 5:**
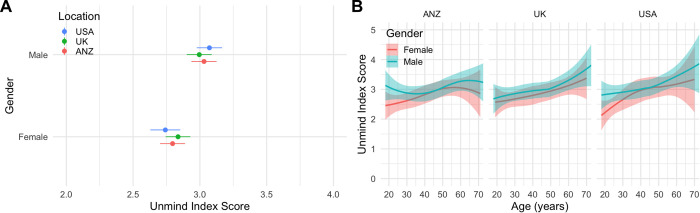
Unmind Index total scores by location, gender, and age, for Study 2. **A.** Means (± standard errors) by location and gender, **B.** LOESS smoothed estimates (± standard errors) by age, gender, and location.

Benchmarks for overall Unmind Index scores by location, gender, and age are shown in Fig 5 and Table 9. Full tables are reported in S1 File. Scores were consistently higher for male participants, and for older participants, but did not differ systematically between locations. Linear model coefficients are reported in Table 10.

**Table 9:** Benchmark means (± standard deviations) of total Unmind Index scores by location, gender, and age group, from Study 2.

		Age group			
Location	Gender	18–25	26–40	41–50	51–83
ANZ	Female	2.56 (±0.89)	2.59 (±0.97)	2.88 (±0.91)	3.14 (±0.90)
ANZ	Male	3.02 (±0.79)	2.85 (±0.89)	3.00 (±1.10)	3.25 (±0.94)
UK	Female	2.55 (±1.02)	2.73 (±0.91)	2.87 (±0.90)	3.16 (±0.83)
UK	Male	2.71 (±1.16)	2.97 (±0.88)	3.01 (±0.82)	3.35 (±0.81)
USA	Female	2.27 (±0.86)	2.68 (±1.00)	3.15 (±1.02)	3.02 (±1.31)
USA	Male	2.82 (±0.87)	3.13 (±0.84)	3.03 (±1.11)	3.39 (±0.79)

**Table 10:** Linear model coefficients for effects of location, gender, and age group on total Unmind Index score, Study 2.

Coefficient	Estimate	SE
Intercept (UK, female, 40)	2.79	0.08
Location = ANZ	0.00	0.09
Location = USA	0.01	0.09
Gender = Male	0.24	0.08
Age (+10 years)	0.15	0.03

### Discussion

These results provide evidence that the Unmind Index total score and the individual subscales display strong measurement invariance across participants from the UK, USA, and ANZ.

## General discussion

Taken together, our results establish that the Unmind Index is an appropriate measure of MHWB in the UK, USA, and Australia/New Zealand. In Study 1, we demonstrated that the second-order factor model of the Unmind Index adequately captures the covariance structure of the 26 items that make up the Unmind Index in USA and ANZ samples. Furthermore, this model yields factor scores almost perfectly correlated with a more complicated model which excellently captures the structure. We also demonstrated good reliability (internal consistent and test-retest reliability) for all seven subscales, and excellent reliability for the total score, in both locations.

Correlations with existing measures of mental health and wellbeing were strong and broadly as expected in both locations. Some correlations with related existing measures were stronger than expected, e.g. between the *Fulfilment* subscale and the WEMWBS, a measure of general mental wellbeing. This suggests that the Unmind Index subscales are not always highly specific measures of MHWB. However, these cross-correlations are commonly found for MHWB measures, and likely reflect the transdiagnostic nature of many psychological attributes [22]. In future work, we hope to further explore this validation from a transdiagnostic perspective.

In Study 1 we also established that the overall mental health and wellbeing score showed evidence of strong measurement invariance by gender and by age group in both locations In Study 2, we found evidence of measurement invariance by location (UK, USA, or ANZ) for overall scores and for all subscales. All subscales displayed strong measurement invariance by gender in both studies, but the *Fulfilment* subscale showed only weak invariance by age group in both studies. Inspection of item means by group (not reported) revealed that this lack of invariance was due to older participants scoring 0.8 points higher on all *Fulfilment* items except for “*[…] felt that I am growing positively as a person*”, on which older and younger participants did not differ. This is perhaps unsurprising, and consistent with recent work showing that the Subjective Happiness Scale, a similar measure, also shows only weak invariance by age [27].

Measurement invariance is a necessary condition for comparing scores across groups; if measurement invariance does not hold, scores cannot be validly compared across groups. It should be noted, however, that it not a sufficient condition for comparison, and there may be other sources of bias in comparing scores from men and women, older or younger users, or users in different locations, that are not captured by these analyses.

We would also note that this study addresses the validity and invariance of the Unmind Index in several Western, English-speaking, and industrialised countries: the UK, USA, Australia, and New Zealand. It may be likely that these results would generalise to similar countries, such as Canada or Ireland. However, further work is required to establish the validity and psychometric characteristics of the Unmind Index in non-Western and non-English-speaking locations. This work is ongoing.

We noted above that it is rare for MHWB measures developed in one English-speaking country to be properly validated for use in other such countries. Our results show that the Unmind Index, developed in the UK, is indeed valid for use in the United States, Australia, and New Zealand. However, it is not yet clear to what extent these results would generalise to other measures of MWHB. We would therefore encourage researchers and practitioners to consider validating the measures they use whenever possible, even if said measures have been validated for use in other English-speaking countries.

This work has a few limitations that should be noted. Since recruitment was carried out using the Prolific platform, the participants sampled were of course limited to users of that platform, and biased towards more active users. We cannot rule unmeasured differences between this sample and the general population. However, this limitation is common to all but the most sophisticated survey studies. Another related limitation is the smaller-than-planned sample size of older participants in Australia/New Zealand. We also note that data collection took place throughout 2021, during the COVID-19 pandemic. Interestingly, previous research [28] has shown that at least one measure of state affect and one measure of trait affect show strict measurement invariance when comparing data from before and during the acute phase of the pandemic. In general, though, it is not known how the psychometric properties of MHWB measures are affected by major events like the COVID pandemic.

To conclude, our results indicate that the Unmind Index is fit for purpose as a multifactor measure of MWHB for users in the UK, United States, Australia and New Zealand. They also indicate no issues in comparing Unmind Index scores across age or gender groups, or across these locations. Unmind as an organisation is committed to providing high-quality MHWB support for users around the world, and these findings indicate that the Unmind Index fulfils part of that goal. Future work will include translation of the Unmind Index into other languages, and validation of these translations.

## Supporting information

S1 File Supplementary figures, tables, details, and analyses relating to the studies reported in this manuscript. (DOCX)
