# Supplementary material for: Validating the Unmind Index as a measure of mental health and wellbeing among adults in USA, Australia, and New Zealand
Source: PLoS One. 2023 Nov 2;18(11):e0287215. doi: 10.1371/journal.pone.0287215 (PMC10621920; doi:10.1371/journal.pone.0287215)
Supplement: S1 File — (DOCX) [file pone.0287215.s001.docx]

Validating the Unmind Index as a measure of mental health and wellbeing among adults in USA, Australia, and New Zealand

Eoin Travers, Unmind Ltd

Bao Sheng Loe, University of Cambridge

Luning Sun, University of Cambridge

Heather Bolton, Unmind Ltd

# Supplementary Materials

## Study 1: Correlation Residuals


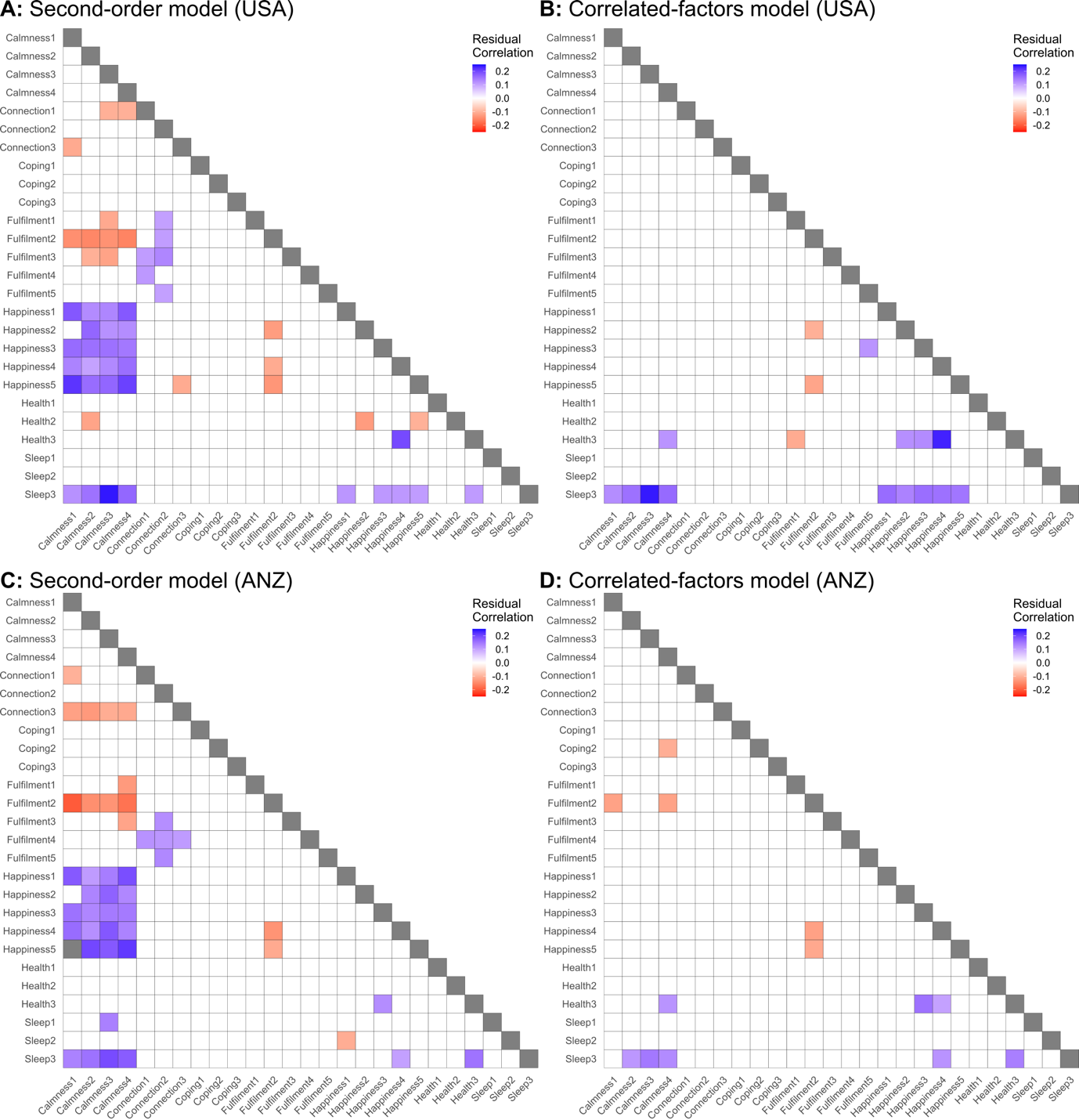


## Figure S1. Correlation residuals greater than 0.1 in absolute value for the second-order (**A, C**) and correlated-factors CFA (**B, D**) models, for the USA (**A, B**) and ANZ (**C, D**) samples. Large residuals reflect ways in which a model fails to fully capture the correlation between pairs of items.

## Study 1: Measurement Invariance

Tables S1 and S2 show the results of the measurement invariance analyses for Study 1, conducted separately for each of the seven Unmind Index subscales. Measurement invariance was assessed by gender and by age group, separately for each location (USA and ANZ). By gender (Table S1), all seven subscales showed evidence of measurement invariance in both locations, with the strong invariance model obtaining lowest BIC scores, and reductions in CFI of less than -.01 in all cases.

By age group (Table S2), there was some evidence that measurement invariance may not hold for the *Coping* subscale among USA participants, as CFI decreased from 1 for the configural invariance model to .983 (Δ=-.017) for the weak invariance model, and .955 (Δ=-.029) for the strong invariance model, and BIC scores not differing appreciably between models (ΔBIC ≤ 4). There was also some evidence that strong measurement invariance may not hold for the *Calmness, Fulfilment*, and *Happiness* subscales in ANZ participants, as the strong invariance models had lower CFI scores (ΔCFI = -.021, -.015, and -.012 points respectively) and only slightly better BICs (ΔBIC = 4, 2, and 8) compared to the weak invariance models.

Table S1: Measurement invariance results by gender (male or female), Study 1.

|  |  |  | Comparative Fit Index | | |  | Bayesian Information Criterion | | |
| --- | --- | --- | --- | --- | --- | --- | --- | --- | --- |
| Territory | Scale |  | Config. | Weak | Strong |  | Config. | Weak | Strong |
| USA | Unmind Index |  | .900 | .899 (Δ=-0.001) | .898 (Δ=-0.002) |  | 194 | 75 (Δ=-119) | 0 (Δ= 75) |
|  | Fulfilment |  | 1.000 | 1.000 (Δ=-.000) | 1.000 (Δ=.000) |  | 39 | 23 (Δ=-16) | 0 (Δ=-23) |
|  | Connection |  | 1.000 | .999 (Δ=-.001) | .990 (Δ=-.009) |  | 14 | 5 (Δ=-9) | 0 (Δ=-5) |
|  | Happiness |  | .989 | .989 (Δ=.001) | .981 (Δ=-.008) |  | 30 | 9 (Δ=-21) | 0 (Δ=-9) |
|  | Health |  | 1.000 | .996 (Δ=-.004) | 1.000 (Δ=.004) |  | 20 | 12 (Δ=-8) | 0 (Δ=-12) |
|  | Coping |  | 1.000 | 1.000 (Δ=-.000) | 1.000 (Δ=.000) |  | 21 | 11 (Δ=-10) | 0 (Δ=-11) |
|  | Sleep |  | 1.000 | 1.000 (Δ=.000) | 1.000 (Δ=.000) |  | 22 | 10 (Δ=-11) | 0 (Δ=-10) |
|  | Calmness |  | 1.000 | 1.000 (Δ=.000) | .996 (Δ=-.004) |  | 27 | 12 (Δ=-15) | 0 (Δ=-12) |
| ANZ | Unmind Index |  | 0.907 | .909 (Δ =.002) | .909 (Δ = -0.000) |  | 226 | 88 (Δ=-137) | 0 (Δ=-88) |
|  | Fulfilment |  | 1.000 | 1.000 (Δ=.000) | 1.000 (Δ=.000) |  | 44 | 23 (Δ=-21) | 0 (Δ=-23) |
|  | Connection |  | 1.000 | 1.000 (Δ=.000) | 1.000 (Δ=.000) |  | 22 | 11 (Δ=-12) | 0 (Δ=-11) |
|  | Happiness |  | .991 | .994 (Δ=.003) | .994 (Δ=.001) |  | 43 | 20 (Δ=-22) | 0 (Δ=-20) |
|  | Health |  | 1.000 | .998 (Δ=-.002) | 1.000 (Δ=.002) |  | 19 | 11 (Δ=-9) | 0 (Δ=-11) |
|  | Coping |  | 1.000 | 1.000 (Δ=.000) | .995 (Δ=-.005) |  | 17 | 7 (Δ=-10) | 0 (Δ=-7) |
|  | Sleep |  | 1.000 | 1.000 (Δ=.000) | 1.000 (Δ=.000) |  | 22 | 11 (Δ=-12) | 0 (Δ=-11) |
|  | Calmness |  | .973 | .972 (Δ=-.001) | .967 (Δ=-.005) |  | 26 | 12 (Δ=-14) | 0 (Δ=-12) |

Table S2: Measurement invariance results by age group (above or below median in each location), Study 1.

|  |  |  | Comparative Fit Index | | |  | Bayesian Information Criterion | | |
| --- | --- | --- | --- | --- | --- | --- | --- | --- | --- |
| Territory | Scale |  | Config. | Weak | Strong |  | Config. | Weak | Strong |
| USA | Unmind Index |  | .890 | .891 (Δ≈0) | .885 (Δ=-.005) |  | 175 | 50 (Δ=-125) | 0 (Δ=-50) |
|  | Fulfilment |  | 1.000 | 1.000 (Δ≈0) | .990 (Δ=-.010) |  | 27 | 5 (Δ=-22) | 0 (Δ=-5) |
|  | Connection |  | 1.000 | 1.000 (Δ≈0) | 1.000 (Δ≈0) |  | 22 | 12 (Δ=-10) | 0 (Δ=-12) |
|  | Happiness |  | .994 | .994 (Δ≈0) | .990 (Δ=-.004) |  | 35 | 15 (Δ=-20) | 0 (Δ=-15) |
|  | Health |  | 1.000 | .998 (Δ=-.002) | .990 (Δ=-.008) |  | 16 | 7 (Δ=-9) | 0 (Δ=-7) |
|  | **Coping** |  | **1.000** | **.983 (Δ=-.017)** | **.955 (Δ=-.029)** |  | **4** | **0 (Δ=-4)** | **1 (Δ=1)** |
|  | Sleep |  | 1.000 | 1.000 (Δ=-.000) | 1.000 (Δ=-.000) |  | 20 | 10 (Δ=-10) | 0 (Δ=-10) |
|  | Calmness |  | .999 | 1.000 (Δ=.001) | .990 (Δ=-.010) |  | 24 | 6 (Δ=-17) | 0 (Δ=-6) |
| ANZ | Unmind Index |  | .897 | .896 (Δ≈0) | .891 (Δ=-.006) |  | 171 | 49 (Δ=-122) | 0 (Δ=-49) |
|  | **Fulfilment** |  | **.992** | **.992 (Δ≈0)** | **.977 (Δ=-.015)** |  | **21** | **2 (Δ=-20)** | **0 (Δ=-2)** |
|  | Connection |  | 1.000 | 1.000 (Δ≈0) | 1.000 (Δ≈0) |  | 21 | 9 (Δ=-11) | 0 (Δ=-9) |
|  | **Happiness** |  | **.987** | **.990 (Δ=.003)** | **.978 (Δ=-.012)** |  | **31** | **8 (Δ=-23)** | **0 (Δ=-8)** |
|  | Health |  | 1.000 | .997 (Δ=-.003) | .995 (Δ=-.001) |  | 17 | 9 (Δ=-8) | 0 (Δ=-9) |
|  | Coping |  | 1.000 | .997 (Δ=-.003) | 1.000 (Δ=.003) |  | 20 | 11 (Δ=-8) | 0 (Δ=-11) |
|  | Sleep |  | 1.000 | .991 (Δ=-.009) | .992 (Δ=.001) |  | 15 | 10 (Δ=-5) | 0 (Δ=-10) |
|  | **Calmness** |  | **.978** | **.980 (Δ=.002)** | **.959 (Δ=-.021)** |  | **20** | **4 (Δ=-16)** | **0 (Δ=-4)** |

## Study 1: Correlations without disattenuation


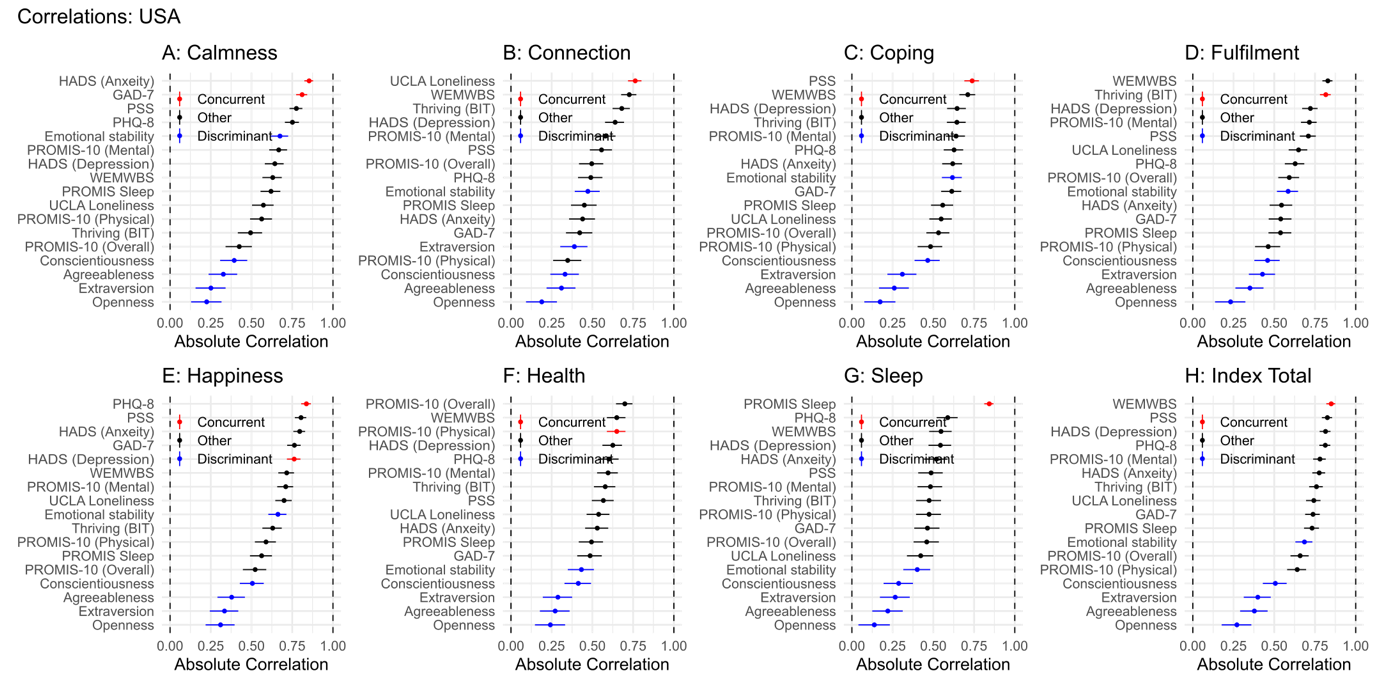


Figure S2. Raw (not dis-attenuated) absolute correlation coefficients between Unmind Index scores and existing measures for the USA sample. Values in red show correlations with mental health and wellbeing measures predicted to correlate most strongly with the Unmind Index subscale in question. Values in blue show personality measures, which were expected to correlate most weakly with all scales. Error bars show standard error.


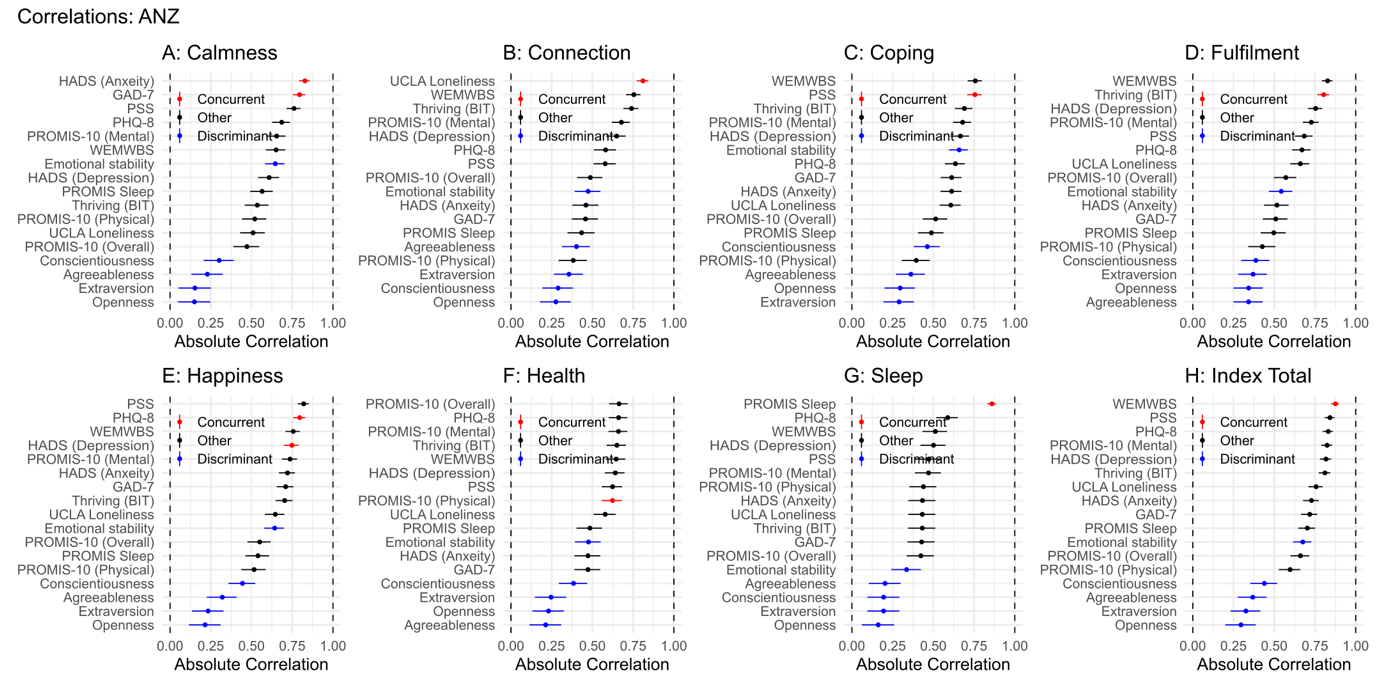


Figure S3. Raw (not dis-attenuated) absolute correlation coefficients between Unmind Index scores and existing measures for the ANZ sample. Values in red show correlations with mental health and wellbeing measures predicted to correlate most strongly with the Unmind Index subscale in question. Values in blue show personality measures, which were expected to correlate most weakly with all scales. Error bars show standard error.

## Study 2: Demographics

Table S3. Breakdown of participant ethnicity by location, Study 2. Participants indicated the ethnicity they identified in a free text field. Responses were recoded into the categories below for reporting purposes.

| Ethnicity | ANZ (N=200) | UK (N=200) | USA (N=198) | Total (N=598) |
| --- | --- | --- | --- | --- |
| European (includes “British”) | 130 (65.0%) | 176 (88.0%) | 116 (58.6%) | 422 (70.6%) |
| Asian | 26 (13.0%) | 6 (3.0%) | 27 (13.6%) | 59 (9.9%) |
| Hispanic | 3 (1.5%) | 0 (0%) | 27 (13.6%) | 30 (5.0%) |
| Australian/New Zealander | 18 (9.0%) | 0 (0%) | 0 (0%) | 18 (3.0%) |
| Mixed | 4 (2.0%) | 6 (3.0%) | 7 (3.5%) | 17 (2.8%) |
| African | 1 (0.5%) | 2 (1.0%) | 12 (6.1%) | 15 (2.5%) |
| South Asian | 8 (4.0%) | 4 (2.0%) | 3 (1.5%) | 15 (2.5%) |
| [OTHER] | 10 (5.0%) | 6 (3.0%) | 6 (3.0%) | 22 (3.7%) |

## Study 2: Measurement Invariance

Table S4. Full measurement invariance results for Study 2. Value show comparative fit indices and Bayesian information criteria calculated for configural (*Config*), weak, and strong invariance CFA models, for both the full Unmind Index model (*Total*) and each subscale, where measurement invariance is estimated as a function of location, age group (above or below median per location), and gender (male or female). Values indicating violations of invariance are shown in bold.

| Group variable | Scale |  | Comparative Fit Index | | | |  | Bayesian Information Criterion | | |
| --- | --- | --- | --- | --- | --- | --- | --- | --- | --- | --- |
|  |  |  | Config | | Weak | Strong |  | Config | Weak | Strong |
| Location | Total |  | .903 | | .904 (Δ=+.001) | .902 (Δ=-.002) |  | 444 | 162 (Δ=-282) | 0 (Δ=-162) |
|  | Fulfilment |  | .974 | | .977 (Δ=+.003) | .975 (Δ=-.002) |  | 89 | 39 (Δ=-50) | 0 (Δ=-39) |
|  | Connection |  | 1.000 | | .999 (Δ=-.001) | 1.000 (Δ=+.001) |  | 44 | 23 (Δ=-21) | 0 (Δ=-23) |
|  | Happiness |  | .992 | | .994 (Δ=+.002) | .992 (Δ=-.002) |  | 87 | 40 (Δ=-47) | 0 (Δ=-40) |
|  | Health |  | 1.000 | | .994 (Δ=-.006) | .991 (Δ=-.003) |  | 37 | 20 (Δ=-17) | 0 (Δ=-20) |
|  | Coping |  | 1.000 | | .997 (Δ=-.003) | .990 (Δ=-.007) |  | 35 | 16 (Δ=-19) | 0 (Δ=-16) |
|  | Sleep |  | 1.000 | | 1.000 (Δ≈0) | .996 (Δ=-.004) |  | 38 | 15 (Δ=-24) | 0 (Δ=-15) |
|  | Calmness |  | .972 | | .976 (Δ=+.004) | .968 (Δ=-.008) |  | 60 | 25 (Δ=-36) | 0 (Δ=-25) |
| Age group | Total |  | .908 | | .907 (Δ=-.001) | .905 (Δ=-.002) |  | 198 | 68 (Δ=-130) | 0 (Δ=-68) |
|  | **Fulfilment** |  | **.979** | | **.978 (Δ=-.001)** | **.965 (Δ=-.013)** |  | **20** | **0 (Δ=-20)** | **6 (Δ=+6)** |
|  | Connection |  | 1.000 | | 1.000 (Δ≈0) | .998 (Δ=-.002) |  | 20 | 8 (Δ=-11) | 0 (Δ=-8) |
|  | Happiness |  | .992 | | .992 (Δ≈0) | .992 (Δ≈0) |  | 44 | 22 (Δ=-22) | 0 (Δ=-22) |
|  | Health |  | 1.000 | | .999 (Δ=-.001) | 1.000 (Δ=+.001) |  | 23 | 13 (Δ=-10) | 0 (Δ=-13) |
|  | Coping |  | 1.000 | | 1.000 (Δ≈0) | .999 (Δ=-.001) |  | 21 | 9 (Δ=-12) | 0 (Δ=-9) |
|  | Sleep |  | 1.000 | | 1.000 (Δ≈0) | 1.000 (Δ≈0) |  | 21 | 9 (Δ=-13) | 0 (Δ=-9) |
|  | Calmness |  | .978 | | .981 (Δ=+.003) | .980 (Δ=-.001) |  | 34 | 16 (Δ=-19) | 0 (Δ=-16) |
| Gender | Total |  | | .907 | .906 (Δ=-.001) | .905 (Δ=-.001) |  | 211 | 93 (Δ=-119) | 0 (Δ=-93) |
|  | Fulfilment |  | .979 | | .980 (Δ=+.001) | .976 (Δ=-.004) |  | 37 | 14 (Δ=-23) | 0 (Δ=-14) |
|  | Connection |  | 1.000 | | .995 (Δ=-.005) | .996 (Δ=+.001) |  | 18 | 11 (Δ=-7) | 0 (Δ=-11) |
|  | Happiness |  | .988 | | .986 (Δ=-.002) | .987 (Δ=+.001) |  | 41 | 24 (Δ=-17) | 0 (Δ=-24) |
|  | Health |  | 1.000 | | .999 (Δ=-.001) | .994 (Δ=-.005) |  | 17 | 7 (Δ=-10) | 0 (Δ=-7) |
|  | Coping |  | 1.000 | | 1.000 (Δ≈0) | 1.000 (Δ≈0) |  | 21 | 10 (Δ=-11) | 0 (Δ=-10) |
|  | Sleep |  | 1.000 | | 1.000 (Δ≈0) | 1.000 (Δ≈0) |  | 25 | 13 (Δ=-13) | 0 (Δ=-13) |
|  | Calmness |  | .976 | | .977 (Δ=+.001) | .977 (Δ≈0) |  | 33 | 16 (Δ=-17) | 0 (Δ=-16) |

## Study 2: Group Comparisons

Table S5. Linear model coefficients (± standard errors) for effects of location, gender, and age group on all Unmind Index subscales.

| **Term** | **Total** | **Happiness** | **Calmness** | **Coping** | **Sleep** | **Health** | **Connection** | **Fulfilment** |
| --- | --- | --- | --- | --- | --- | --- | --- | --- |
| Intercept  (UK, female, 40) | 2.79 (±0.08) | 2.81 (±0.10) | 2.41 (±0.10) | 3.12 (±0.08) | 2.74 (±0.11) | 2.59 (±0.10) | 3.11 (±0.09) | 2.76 (±0.09) |
| Location = ANZ | -0.00 (±0.09) | -0.00 (±0.12) | 0.17 (±0.12) | 0.00 (±0.10) | -0.07 (±0.13) | -0.04 (±0.12) | -0.13 (±0.11) | 0.04 (±0.11) |
| Location = USA | 0.01 (±0.09) | -0.04 (±0.12) | -0.01 (±0.13) | 0.01 (±0.10) | 0.00 (±0.14) | 0.09 (±0.12) | -0.05 (±0.11) | 0.09 (±0.11) |
| Gender = Male | 0.24 (±0.08) | 0.20 (±0.10) | 0.52 (±0.10) | 0.30 (±0.08) | 0.16 (±0.11) | 0.22 (±0.10) | 0.18 (±0.09) | 0.09 (±0.09) |
| Age (+10 years) | 0.15 (±0.03) | 0.25 (±0.03) | 0.22 (±0.03) | 0.21 (±0.03) | 0.05 (±0.04) | 0.11 (±0.03) | 0.10 (±0.03) | 0.13 (±0.03) |

Table S6. Mean scores (± standard errors) for each Unmind Index subscale, by location.

| **Location** | **N** | **Calmness** | **Connection** | **Coping** | **Fulfilment** | **Happiness** | **Health** | **Sleep** | **Total** |
| --- | --- | --- | --- | --- | --- | --- | --- | --- | --- |
| ANZ | 198 | 2.85 (±0.09) | 3.08 (±0.08) | 3.28 (±0.07) | 2.85 (±0.08) | 2.92 (±0.09) | 2.67 (±0.08) | 2.75 (±0.10) | 2.91 (±0.07) |
| UK | 200 | 2.68 (±0.09) | 3.21 (±0.08) | 3.27 (±0.07) | 2.81 (±0.08) | 2.92 (±0.09) | 2.70 (±0.09) | 2.82 (±0.09) | 2.92 (±0.07) |
| USA | 194 | 2.64 (±0.10) | 3.15 (±0.09) | 3.25 (±0.08) | 2.88 (±0.08) | 2.85 (±0.10) | 2.77 (±0.09) | 2.81 (±0.10) | 2.91 (±0.07) |

Table S7. Mean scores (± standard errors) for each Unmind Index subscale, by gender.

| **Gender** | **N** | **Calmness** | **Connection** | **Coping** | **Fulfilment** | **Happiness** | **Health** | **Sleep** | **Total** |
| --- | --- | --- | --- | --- | --- | --- | --- | --- | --- |
| Female | 297 | 2.46 (±0.07) | 3.05 (±0.07) | 3.12 (±0.06) | 2.80 (±0.07) | 2.79 (±0.07) | 2.61 (±0.07) | 2.71 (±0.08) | 2.79 (±0.06) |
| Male | 295 | 2.98 (±0.08) | 3.24 (±0.06) | 3.42 (±0.06) | 2.89 (±0.06) | 3.00 (±0.07) | 2.82 (±0.07) | 2.87 (±0.08) | 3.03 (±0.05) |

Table S8. Mean scores (± standard errors) for each Unmind Index subscale, by age group.

| **Age** | **N** | **Calmness** | **Connection** | **Coping** | **Fulfilment** | **Happiness** | **Health** | **Sleep** | **Total** |
| --- | --- | --- | --- | --- | --- | --- | --- | --- | --- |
| 18 - 25 | 151 | 2.25 (±0.10) | 2.96 (±0.09) | 2.89 (±0.08) | 2.62 (±0.09) | 2.42 (±0.10) | 2.54 (±0.09) | 2.71 (±0.11) | 2.63 (±0.08) |
| 26 - 40 | 145 | 2.63 (±0.10) | 3.06 (±0.09) | 3.12 (±0.09) | 2.74 (±0.09) | 2.75 (±0.09) | 2.73 (±0.10) | 2.82 (±0.10) | 2.84 (±0.08) |
| 41 - 50 | 154 | 2.90 (±0.10) | 3.25 (±0.08) | 3.41 (±0.08) | 2.91 (±0.09) | 3.06 (±0.11) | 2.66 (±0.10) | 2.74 (±0.11) | 2.99 (±0.08) |
| 51 - 83 | 142 | 3.12 (±0.11) | 3.31 (±0.10) | 3.67 (±0.08) | 3.12 (±0.09) | 3.37 (±0.10) | 2.95 (±0.11) | 2.91 (±0.12) | 3.21 (±0.08) |
